# CEP162: A critical regulator of ciliary transition zone assembly and its implications in ciliopathies

**DOI:** 10.1002/ccs3.70012

**Published:** 2025-04-23

**Authors:** Jun Yin, Jialian Bai, Xiaochong He, Wenjuan He, Hongming Miao, Mengjie Zhang, Zhongying Yu, Bing Ni

**Affiliations:** ^1^ Department of Pathophysiology College of High Altitude Military Medicine Army Medical University Chongqing China; ^2^ Key Laboratory of Extreme Environmental Medicine Ministry of Education of China Chongqing China; ^3^ Key Laboratory of High Altitude Medicine PLA Chongqing China; ^4^ School of Artificial Intelligence and Big Data Chongqing Industry Polytechnic College Chongqing China; ^5^ Department of Nursing Administration Faculty of Nursing Army Medical University, (Third Military Medical University) Chongqing China; ^6^ Department of Urology The 909th Hospital School of Medicine Xiamen University Zhangzhou China

**Keywords:** CEP162, cilia, flagella, transition zone

## Abstract

CEP162, a 162‐kDa centrosome protein, is a crucial centrosomal adapter, mediating cell differentiation and polarization. CEP162 maintains mitosis by dynamically stabilizing microtubules. CEP162 promotes the transition zone (TZ) assembly in the basal body through interaction with CEP131, CEP290, and axoneme microtubules as well as the distal centriole. TZ ensures the normal distribution of soluble proteins between the cytoplasm and cilia. It also facilitates retinal development and sperm flagellar motility. However, fluctuations in TZ permeability caused by abnormal expression of CEP162, including truncated mutations and naturally occurring mutations, lead to cilia abnormality and dysfunction in ciliogenesis through the regulation of intraflagellar transport, resulting in retinal degeneration and infertility. LncRNAs can induce SNP events in the CEP162 transcript by altering alternative splicing. Naturally occurring mutations are closely linked to retinal ciliopathy and diabetic retinopathy. This review summarizes the latest research progress to better understand the biology and pathophysiology of CEP162 and the clinical manifestations caused by CEP162 variants.

## Graphical abstract

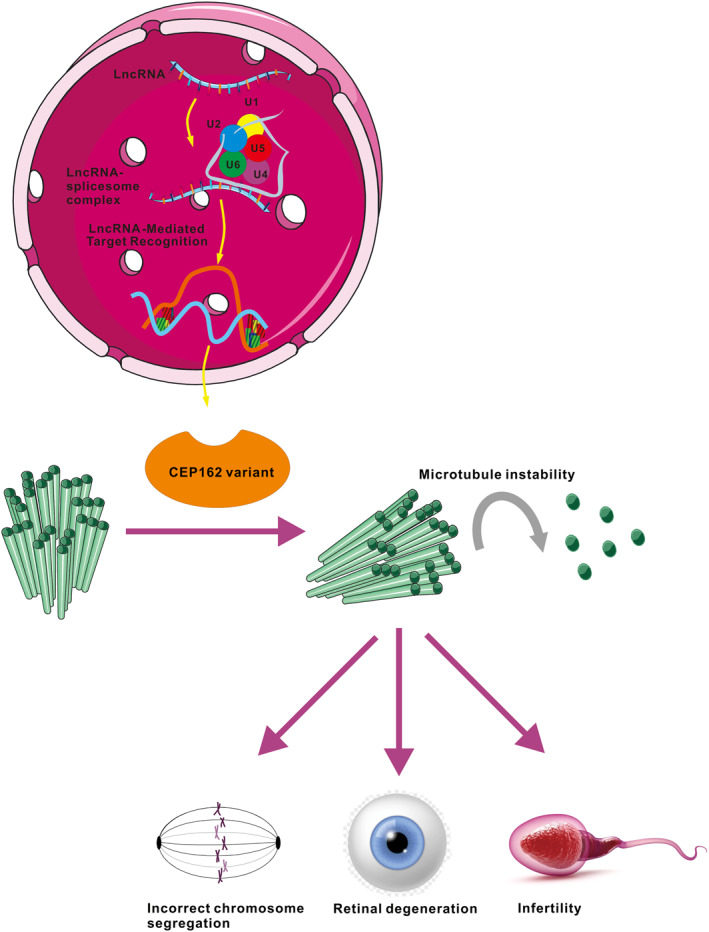

## 1 INTRODUCTION

A mature centrosome consists of a pair of centrioles surrounded by pericentriolar material (PCM). A mature centriole is a cylindrical structure with rotational symmetry consisting of nine triplet microtubules. The triplet microtubule is connected to the cartwheel by the pinhead. The nine triplet microtubules constitute the main organization unit for the centriole and form spindles or basal bodies made up of cilia and flagella. The microtubule is involved in various cellular processes, including cell division, cell motility, intracellular organization, and organelle transport; the establishment of cell polarity and adhesion; and the maintenance of cell morphology.^1^ The absence of microtubules is implicated in serious ciliopathies such as microcephaly, polycystic kidney disease (PKD), nephronophthisis, retinitis pigmentosa, Bardet–Biedl syndrome (BBS), Joubert syndrome, and Meckel syndrome. In the ciliopathies mentioned above, the microtubule‐based structure of the cilia is altered. Additionally, its sensory function may be altered.^2^ Microtubules can alternate between growth and shrinkage phases, and microtubules' growth and shortening dynamics are important for various cell functions. Microtubule‐associated proteins (MAPs) maintain the aforementioned cellular processes by regulating stability and microtubule dynamics.

MAPs broadly apply to any protein that cosediments with microtubules through multiple rounds of polymerization and depolymerization.^3^ MAPs enable microtubules to participate in various cellular processes, such as cell cycle progression, spindle kinetochore assembly, centriole duplication, cell polarity, cell cycle checkpoint signaling mechanisms, neuronal development, and ciliary axoneme formation.^4^ The most important function of MAPs is the nucleation and organization of microtubules and, thus, the coordination of microtubule‐dependent cellular processes. MAPs can regulate stability and microtubule dynamics in several ways (Table 1): (i) A group of MAPs, such as AKNA and CAMSAPs, is involved in microtubule nucleation.^5^, ^21^ (ii) The tubulin polyglutamylase complex subunit L1/7 of the TTL family (TTLL1/7, a group of MAPs) causes tubulin polyglutamylation and increases the degree of microtubule severity associated with katanin.^22^ (iii) MAPs, such as the StAR‐related lipid transfer domain containing 9 (STARD9), can regulate the balance between soluble and polymeric tubulin.^23^ (iv) Microtubule‐associated motor proteins, such as spastin (a type of AAA‐ATPase), can alter the growth/catastrophe frequency of microtubules by severing them upon binding.^24^ Kinesin‐5 (a type of GTPase) pauses at microtubule plus ends and enhances polymerization by stabilizing longitudinal tubulin–tubulin interactions.^25^ (v) MAPs can influence other microtubule‐associated proteins' amount, type, and activity. For example, CEP110 alters the localization of HOOK2, an important protein that influences microtubule nucleation.^19^ CEP162 is required to correctly localize the C‐terminus of CEP290 to the axoneme in spermatocyte cilia. CEP131 interacts with CEP162 and recruits it to the centriole tips and transition zone (TZ).^26^

**TABLE 1:** Highlights of research findings on the effects of MAPs on microtubule dynamics.

Name	Other names	Gene ID	Function related to microtubules	Underlying mechanisms
AKNA^5^	KIAA1968	ENSG00000106948	Promotes centrosomal microtubule nucleation by recruiting γTuRC, faster microtubule growth	Changing microtubule nucleation
CAMSAP2^6^	CAMSAP1L1	ENSG00000118200	Anchors and stabilizes the minus ends of the microtubules	
CCDC66^7^	RGD1560873	ENSG00000180376	Recruits gamma‐tubulin to the centrosomes and spindle	
TPX2^8^	C20orf1	ENSG00000088325	Facilitates branched microtubule nucleation by γ‐tubulin ring complex and TPX2	
ATAT1^9^	C6orf134	ENSG00000137343	Influences kinesin‐1‐mediated organelle displacement to regulate intracellular organization	Post‐translationally modifying tubulin
HNRNPA2B1^10^	HNRNPA2	ENSG00000122566	Links oligomeric tau to N6‐methyladenosine (m6A)‐modified RNA transcripts and increases oligomeric tau‐induced neurodegeneration	
TTLL1^11^	C22orf7	ENSG00000100271	Produces tubulin polyglutamylation and specifically modulates the activities of three key microtubule interactors: the microtubule‐associated protein tau, the microtubule‐severing enzyme katanin, and the molecular motor kinesin‐1	
CEP169^12^	NCKAP5L	ENSG00000167566	Stabilizes microtubules and induces the formation of long microtubule bundles with intensive microtubule acetylation	
ARL2^13^	ARFL2	ENSG00000213465	Functions as a catalytic chaperone that enhances the assembly and maintenance of soluble αβ‐tubulin to support microtubule dynamics	Converting the equilibrium between polymerized and soluble tubulin
STARD9^14^	KIF16A	ENSG00000159433	Promotes the construction of microtubules and increases the ratio of polymeric to soluble tubulin	
CLASP2γ^15^	mKIAA0627	ENSMUSG00000033392	Specificially modulates the switching transitions between microtubule growth and shrinkage without affecting global growth and shrinkage rates	Altering growth of catastrophe frequency
CEP20^16^	FOR20	ENSG00000133393	Reduces the microtubule growth rate and increases the depolymerization rate and catastrophe frequency	
CEP131^17^	AZ1	ENSG00000141577	Recruits CEP162 to centriole tips and TZ and promotes binding of the C‐terminus of CEP290 to the axoneme	Changing microtubule‐associated protein amount, activity, and localization
FAM92^17^, ^18^	CG6405	LOC100879052	Required for the association of the N‐terminus of CEP290 with the ciliary membrane	
CEP110^19^	CP110	ENSG00000103540	The distribution of endogenous HOOK2 is altered, leading to the loss of microtubule radial organization	
CEP162^20^	KIAA1009	ENSG00000135315	Involved in microtubule recognition and mediates CEP290, which is associated with microtubules	

Abbreviations: AKNA, AT‐hook transcription factor; ARL2, ADP‐ribosylation factor‐like GTPase 2; ATAT1, alpha‐tubulin acetyltransferase 1; CAMSAP2, calmodulin‐regulated spectrin‐associated protein family member 2; CCDC66, coiled‐coil domain containing 66; CEP20, centrosomal protein 20; CEP110, centrosomal protein 110; CEP131, centrosomal protein 131; CEP162, centrosomal protein 162; CEP169, centrosomal protein 169; CLASP2γ, CLIP‐associating protein 2; HNRNPA2B1, heterogeneous nuclear ribonucleoprotein A2/B1; STARD9, StAR‐related lipid transfer domain containing 9; TPX2, targeting protein for Xenopus kinesin‐like protein 2; TTLL1, TTL family tubulin polyglutamylase complex subunit L1.

Among these members of MAPs, CEP162 has not been studied extensively, but more and more data demonstrate that CEP162 plays pivotal effects biologically and pathophysiologically,^20^, ^27^ suggesting it is a potential target for related diseases. CEP162 is a member of the kinesin family of microtubule‐associated motor proteins and a microtubule‐activated ATPase and is highly conserved in eukaryotes, including fungi, zebrafish, rats, mice, and humans. A highly conserved motor domain containing approximately 340 amino acid residues was identified at the amino terminus of CEP162. This domain is now known to be shared by a broad superfamily of kinesin‐related proteins.^28^ *CEP162* was initially cloned from retinal neurons and identified on human chromosome 6, which participates in mitotic chromosome separation, microtubule polymerization, and other processes by hydrolyzing ATP.^29^ CEP162 transports cargo anterogradely toward the plus end of microtubules.^30^ In this review, we elucidate the biological function of CEP162, as well as its roles in diseases and the possible underlying mechanisms based on the latest research literature.

AKNA, AT‐hook transcription factor; ARL2, ADP‐ribosylation factor‐like GTPase 2; ATAT1, alpha‐tubulin acetyltransferase 1; CAMSAP2, calmodulin‐regulated spectrin‐associated protein family member 2; CCDC66, coiled‐coil domain containing 66; CEP20, centrosomal protein 20; CEP110, centrosomal protein 110; CEP131, centrosomal protein 131; CEP162, centrosomal protein 162; CEP169, centrosomal protein 169; CLASP2γ, CLIP‐associating protein 2; HNRNPA2B1, heterogeneous nuclear ribonucleoprotein A2/B1; STARD9, StAR‐related lipid transfer domain containing 9; TPX2, targeting protein for Xenopus kinesin‐like protein 2; TTLL1, TTL family tubulin polyglutamylase complex subunit L1.

## 2 BIOLOGICAL FUNCTIONS OF CEP162

### 2.1 CEP162 protein primary structure

**FIGURE 1:**
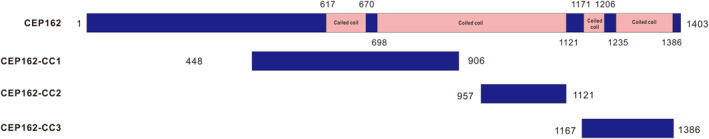
Domain analyses of CEP162 for its CEP131, CEP290, microtubule binding, and centriolar localization. The coiled‐coil domain is indicated in the CEP162 protein. This figure was created using CorelDRAW.

*CEP162* contains 22 transcripts, and the CEP162 protein is expressed mainly in the testis, thyroid, and other tissues.^31^ CEP162 binds to microtubule spindles during mitosis and localizes to the distal ends of centrioles in postmitotic cells. The subcellular localization of CEP162 is mainly in axonemal microtubules, centrioles, centrosomes, and spindles. CEP162 is involved in mitosis, ciliogenesis, transition zone (TZ) assembly, and microtubule dynamics. As shown in Figure 1, full‐length CEP162 (amino acids 1–1403) contains 3 coiled‐coil (CC) stretches in the carboxy‐terminal region: CC1 (amino acids 448–906), CC2 (amino acids 957–1121), and CC3 (amino acids 1167–1386). The function of the CC3 region is to bind the distal centriole (also known as the basal body), whereas the region composed of CC1 and CC2 functions to bind microtubules, CEP131, and CEP290.^20^, ^26^

The CC1 residue of CEP162 interacts with both the N‐terminal half (aa 1–549) and C‐terminal half (aa 550–1114) of CEP131.^26^ The CC1 and CC2 residues of CEP162 initially bind to the C‐terminus of CEP290, and the CEP162‐CEP290 module subsequently associates with many TZ proteins, including CEP290, NPHP1‐4‐8, TCTN1, TMEM67, and NPHP8/RPGRIP1L, which are key biological processes in TZ assembly. The TZ associates with axoneme microtubules and serves as a diffusion barrier between the basal body and the cilia/flagellar membrane, which is used for maintaining the distribution of soluble proteins in the cilia/flagellar and head regions (including the cytoplasm, nucleus, and cell membrane).^30^ In addition, the CC1 and CC2 residues of CEP162 bind to microtubules, and the CC1 and CC2 residues promote mitotic spindle separation and ciliogenesis by maintaining microtubule dynamic stability. The CC3 residue of CEP162 binds to the distal centriole and maintains the spatial limitations of the TZ in the ciliary basal body and the distribution of soluble proteins, including TZ proteins, inside and outside the cell. The interactions between CEP162 and the distal centriole, microtubules, CEP131, and CEP290 are essential for maintaining different cellular functions mediated by different organelles, including cell division and retinal development.^28^ We discuss the effects of CEP162 on different organelles below.

### 2.2 Spindle

Recently, several studies have demonstrated that CEP162 promotes chromosome segregation and mitotic spindle assembly through the maintenance of the stability and dynamics of microtubules in the spindle.^29^ CEP162 retains microtubule binding at the mitotic spindle, allowing it to function during cell division and proliferation. Nuzhat et al. reported that the naturally occurring CEP162 protein mutation maintains microtubule binding and is present at mitotic spindles in patient‐derived fibroblasts. These cells also have a normal complement of chromosomes and normal growth rates.^26^ Wang et al. reported that CEP162 is located on spindle microtubules in retinal pigment epithelial (RPE1) cells during mitosis, where it possesses microtubule‐binding activity and can promote mitotic spindle chromosome separation.^20^ Interference with CEP162 has been verified to inhibit proper neurogenesis and mitosis by hindering spindle separation. Nuzhat et al. demonstrated that the knockdown of CEP162 in the developing mouse retina increased neuronal cell death, which was rescued by the expression of not only full‐length but also truncated CEP162 protein (the truncated CEP162 protein retains residues that bind to microtubules).^26^ Similarly, A. Leon et al. demonstrated the participation of the QN1 (KIAA1009/CEP162) protein in spindle formation and chromosome segregation in NGF‐treated PC12 cells. Transfection with QN1 siRNA led to defects in spindle separation and microtubule polymerization, resulting in incorrect chromosome segregation and the death of PC12 cells.^29^

### 2.3 Cilia

#### 2.3.1 Static cilia

The CEP162‐CEP290 module recruits TZ proteins, such as CEP290 and NPHP4, to form TZs in the basal body, thereby maintaining the distribution and balance of soluble proteins in the cytoplasm and cilia, ultimately promoting ciliogenesis.^32^ During ciliogenesis, the CEP162 signal becomes disc‐shaped and is localized at the ciliary basal body.^20^ Wu et al. reported that CEP162 is a CEP131‐interacting protein that acts downstream of CEP131 to mediate the association of the CEP290 C‐terminus with axonemal microtubules.^17^ In RPE1 cells, siRNA depletion of CEP162 prevented the recruitment of various TZ proteins, blocking the initiation of ciliogenesis.^20^ The truncated CEP162 protein (which binds microtubules but is unable to associate with the distal centriole or CEP290) is expressed and properly localized in the mitotic spindle but is absent in the basal body in the primary and photoreceptor cilia. The truncated CEP162 protein cannot recruit transition zone components to the basal body, which is reflected in the delayed formation of dysmorphic cilia. Interestingly, the overexpression of truncated forms of CEP162 (the truncated CEP162 protein binds CEP290 and microtubules but is unable to associate with the distal centriole) resulted in the distal accumulation of TZ proteins, including CEP290, TMEM67, TCTN1, and RPGRIP1L, at the ciliary tip. However, many TZ proteins, such as CEP290 and RPGRIP1L; intraciliary transport (ICT) markers, such as IFT88; and the ciliary membrane marker ARL13b, detach from the ciliary tips and scatter among retinal pigment epithelial cells. This generates extra‐long cilia with strikingly swollen tips.^20^ Together, these observations demonstrate that the spatial restriction of TZ assembly to the ciliary basal body is critical for proper cilia structure and function. CEP162 is a critical promoter for transition zone assembly by providing such spatial cues.

#### 2.3.2 Motile cilia

Flagella are a type of motile cilia. Flagella and cilia are structurally the same organelles, and flagella are long, motile cilia that generally refer to the cilia of sperm and protists. In *Drosophila* spermatids, axoneme elongation is accompanied by TZ assembly from the distal end of the centriole. As the axoneme is assembled, the TZ continuously migrates in close association with the axoneme's growing end. Consistently, the *Drosophila* ortholog of CEP162 (CG42699) is enriched in the *Drosophila* testis, and multiple sequence alignments of mammalian CEP162 and CG42699 revealed that *Drosophila* does not seem to contain the amino‐terminal region CEP162, which is essential for centriole binding in mammals.^33^ Zhimao Wu et al. demonstrated that the signal of an overexpressed CEP290 carboxyl‐terminus (GFP marker) in CEP162 mutant spermatocytes was significantly lower than that in WT spermatocytes. CEP162 promotes the association between the CEP290 carboxyl‐terminus and microtubules, which contributes to ciliogenesis in spermatocyte flagella.^17^

Compositional differences in the TZ have also been reported between various fly and mouse spermatids and may contribute to the morphological and functional diversity of flagella.^34^ The finding that mutations in TZ components cause leakage of various proteins into and out of the flagellum first established that the TZ is a diffusion barrier for flagellar proteins.^35^ Jungyeon Won et al. reported that sperm were rarely found in the caudal epididymis of male NPHP4 (CEP162‐interacting protein [TZ protein]) mutants (chemically induced mutants, nonsense mutations in exon 4 of Nphp4), and the sperm displayed poor motility and failed to induce fertilization during in vitro fertilization (IVF) experiments.^32^ Veronica Persico et al. reported that mutations in KINESIN‐13 (CEP162‐interacting protein, TZ protein) result in defects in centriole/CLR organization in spermatocytes and flagellar cap assembly in elongating *Drosophila* spermatids.^36^

**FIGURE 2:**
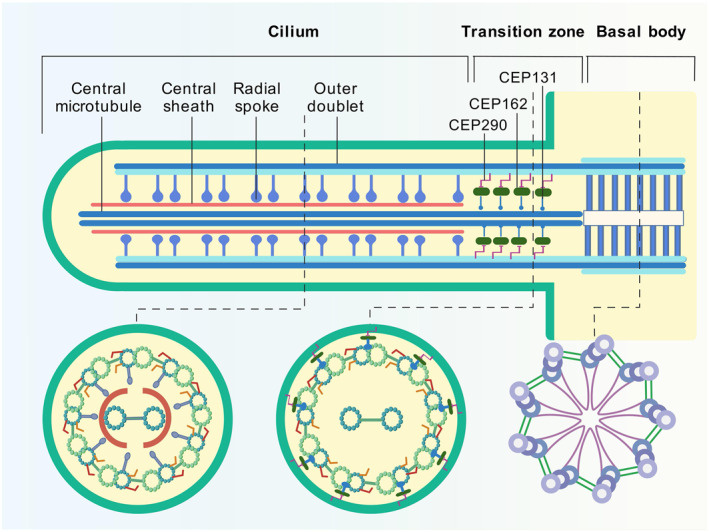
Role of CEP162 in the TZ assembly and protein interactions. Schematic diagram depicting the localization of CEP162 within the TZ of eukaryotic cilia/flagella. The TZ is shown as a structural intermediate between the basal body and the axoneme. CEP162 (green) is highlighted in the TZ, where it interacts with key proteins such as CEP290 (red) and CEP131 (blue) to regulate TZ assembly and microtubule dynamics. The interactions are depicted with dashed lines, emphasizing the coordinated role of these proteins in maintaining cilium integrity. This figure was created using templates and elements from Figdraw (www.figdraw.com).

In summary, as shown in Figure 2, the interaction between CEP162 and CEP131, CEP290, microtubules, and distal centrioles is a crucial promoter for TZ assembly in the basal body, which maintains the distribution of soluble proteins in flagella, as well as the balance of soluble proteins in the cytoplasm and cilia/flagella, thereby maintaining the morphology and function of spermatids. CEP162 deficiency includes truncation mutations that cannot bind to distal centrioles but retain CEP290 and axoneme microtubules, as well as truncation mutations that cannot bind to the distal centrioles and CEP290 but retain axoneme microtubules. CEP162 deficiency, caused by a truncation mutation in the residue of CEP162 that binds to the distal centriole, promotes cilia abnormality in the retinal epithelial cell. CEP162 deficiency, caused by a truncation mutation of the binding residue between CEP162 and the distal centriole and CEP290, can lead to the delayed formation of dysmorphic primary or photoreceptor cilia.

### 2.4 The interplay of CEP162 with IFT proteins

Understanding the role of CEP162 in the context of centrosome and ciliary/flagellar function necessitates an examination of its relationship with IFT proteins. As detailed in recent reviews,^37^, ^38^ IFT proteins are pivotal in connecting ciliary function to centrosome dynamics. Here, we explore how CEP162 processes interface with IFT proteins to modulate cellular function.

CEP162 has been implicated in maintaining the structural integrity of cilia and flagella, which are critical for sensory reception and motility. Its interaction with IFT proteins is crucial for the transport of various cargoes along the ciliary axoneme. Disruptions in this transport mechanism can lead to ciliary dysfunction, which is often associated with CEP162 mutations. The precise nature of these interactions and their implications for cellular physiology are areas of ongoing research.

Our study contributes to the existing body of knowledge by elucidating how CEP162 contributes to the broader cellular mechanisms involving IFT proteins. By contextualizing CEP162 within this framework, we aim to provide a more nuanced understanding of its role in health and disease.

## 3 CEP162‐RELATED DISEASES

CEP162 is a key regulator of the TZ in eukaryotic cilia/flagella, which acts as a structural intermediate between the basal body and the axoneme. The TZ is crucial for regulating ciliary trafficking and maintaining cilium integrity. Mutations in genes encoding TZ proteins (TZPs) are associated with a spectrum of human inherited diseases collectively termed ciliopathies.^39^, ^40^, ^41^ These diseases present with diverse clinical phenotypes, including retinal degeneration, intellectual disabilities, and male infertility. Given the critical role of CEP162 in TZ formation, its abnormal expression or function can lead to altered TZ permeability and the development of ciliopathies.

Retinal degeneration is one of the most studied ciliopathies associated with CEP162 mutations. Nuzhat et al. reported that truncated mutations in CEP162 result in a loss of its ability to bind to the basal body in primary and photoreceptor cilia, leading to retinal impairment.^26^ Similarly, Wang et al. demonstrated that loss of CEP162 function arrests ciliogenesis at the transition zone assembly stage, causing a ciliopathy phenotype in zebrafish.^20^ Awata et al. found that dysregulation of the CEP162 gene is associated with susceptibility to diabetic retinopathy (DR) in Japanese patients with type 2 diabetes,^42^ whereas Li et al. reported a similar association between CEP162 gene variants and nonproliferative DR in a Han Chinese population.^27^ Jungyeon Won observed that in NPHP4 (a TZ protein and CEP162‐interacting protein) mutant retinas, photoreceptor outer segments (OS) fail to develop properly, with some OS markers mislocalizing to the inner segments and outer nuclear layer.^32^ These findings highlight the critical role of CEP162 in maintaining retinal cilia function and preventing retinal degeneration.

In addition to retinal degeneration, CEP162 is also implicated in male infertility through its role in regulating the distribution of microtubule inner proteins (MIPs) in sperm flagella. Jun Wang et al. showed that knocking out RPGRIP1L (a TZ marker and CEP162‐interacting protein) in embryos via CRISPR/Cas9 leads to flagellar phenotypes associated with infertility.^43^, ^44^, ^45^ CEP162 interacts with core TZ components, such as CEP290, and mediates their association with microtubules. Abnormal expression of CEP162 is speculated to disrupt the distribution of MIPs, leading to asthenozoospermia. Emma A. Hall et al. reported that mutations in Azi1 (another TZ marker and CEP162‐interacting protein) result in complete infertility in male mice, characterized by reduced testis weight, decreased sperm density, and severe flagellar defects.^46^ Similarly, Mingrong Lv et al. found that mutations in DZIP1 (a TZ marker and CEP162‐interacting protein) cause male infertility in humans, characterized by asthenoteratospermia and defects in both flagellar formation and sperm centrioles.^47^ These studies suggest that fluctuations in TZ permeability, caused by abnormal CEP162 expression, can lead to an abnormal distribution of soluble proteins in cilia via intraflagellar transport, ultimately resulting in infertility.

Beyond retinal degeneration and infertility, CEP162 mutations have also been associated with other ciliopathies, including Joubert syndrome, characterized by cerebellar ataxia and intellectual disability, and Meckel–Gruber syndrome, which presents with severe developmental abnormalities.^48^, ^49^ Additionally, CEP162 has been implicated in Bardet–Biedl syndrome (BBS) and nephronophthisis, both of which are associated with renal dysfunction and cystic kidneys.^17^, ^50^ The diverse clinical manifestations of these ciliopathies highlight the multifaceted role of CEP162 in maintaining ciliary function and cellular homeostasis.

**FIGURE 3:**
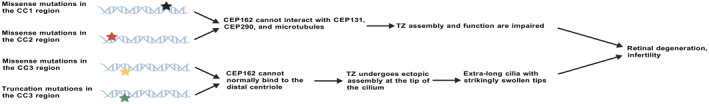
Impact of CEP162 mutations on protein function and disease mechanisms. The diagram illustrates different types of mutations, including truncated mutations and missense mutations. These mutations are shown to disrupt the structural integrity of CEP162, impairing its ability to bind to the basal body and interact with other critical proteins. Specifically, missense mutations in the CC1 and CC2 regions can disrupt interactions with CEP131, CEP290, and microtubules, leading to impaired TZ assembly and ciliary dysfunction. Truncation and missense mutations within the CC3 region can prevent normal binding to the distal centriole, resulting in mislocalization of the TZ and structural abnormalities in cilia. The process begins with genetic mutations that disrupt the normal function of CEP162, resulting in impaired assembly of the transition zone (TZ) and subsequent ciliary dysfunction. This disruption manifests in phenotypes such as retinal degeneration, characterized by photoreceptor cilia abnormalities, and infertility, marked by defects in sperm flagella. This figure was created using templates and elements from Figdraw (www.figdraw.com).

In summary, the involvement of CEP162 in a range of ciliopathies underscores its critical role in ciliary function and cellular homeostasis. As shown in Figure 3, abnormal expression or function of CEP162 can lead to altered TZ permeability and disrupted ciliary trafficking, resulting in diseases such as retinal degeneration and infertility. Understanding the molecular mechanisms underlying these pathophysiological conditions is essential for developing targeted therapies and improving patient outcomes.

## 4 REGULATION OF CEP162

SNPs can be observed between individuals in a population and can influence, for example, promoter activity (gene expression), messenger RNA (mRNA) conformation (stability), and translation efficiency. Mutations during DNA replication, repair, and recombination, and noncoding RNAs, especially long noncoding RNAs (lncRNAs), affect alternative splicing (AS) and generate alternative splicing isoforms, resulting in SNPs. LncRNAs mainly regulate pre‐mRNAs' AS in three ways: (i) LncRNAs regulate AS through cis‐acting elements. LncRNAs can specifically interact with cis‐acting elements in pre‐mRNAs through RNA–RNA base pairing. The interaction between lncRNAs and cis‐acting elements can influence the selection of splice sites and the recruitment of splicing factors (SFs), regulating the expression of alternatively spliced ​​isoforms. (ii) LncRNAs regulate AS through trans‐acting factors. LncRNAs are involved as post‐transcriptional regulators of the mRNA expression of SFs, influencing splicing isoform outcomes. (iii) LncRNAs regulate AS through pre‐mRNA transcription. LncRNAs can regulate AS, such as exon exclusion, by influencing transcription, mainly through interactions with Pol II and the regulation of histone modifications and DNA methylation.^51^

A strong correlation was found between the CEP162 SNPs and retinopathy in diabetic retinopathy (DR) and type 2 diabetes mellitus. Nonsynonymous SNPs (changing amino acids, CEP162 gene variant) can often lead to retinal diseases and are therefore subject to natural selection.^52^ In a GWAS of Japanese DR patients, the SNP with the top signal from the association analysis was rs9362054 in an intron of RP1‐90L14.1 (a long intergenic noncoding RNA/lincRNA) adjacent to the CEP162 gene. rs9362054 is significantly associated with diabetic nephropathy (DN) independent of the severity of DR.^42^ Li et al., in a total of 957 individuals with type 2 diabetes mellitus (T2DM), including diabetes mellitus without retinopathy (DNR = 478), nonproliferative DR (NPDR = 384), and proliferative DR (PDR = 95), reported that there was a statistically significant difference in CEP162 rs9362054 between NPDR and DNR. For rs9362054, the TT genotype was much more common in patients with NPDR than in those with DNR (12.9% vs. 10.1%), demonstrating a risk effect of the T allele. A significant association of rs9362054 was detected under the dominant model in the NPDR group (TT + CT/CC, *p* = 0.03, OR = 1.35, 95% CI = 1.03–1.77) compared with the DNR group, indicating that subjects carrying the TT + CT genotype were more likely to suffer from NPDR for rs9362054.^27^ Previous reports have shown that lncRNAs may be involved in alternative polyadenylation (APA) interactions by miRNAs and, thus, in the generation of SNPs (single nucleotide polymorphisms, normal variations in the human genome, and mRNA isoforms with extended 3′‐UTRs) in the CEP162 transcript.^51^, ^53^ Overall, CEP162 SNPs are associated with the development of DR and diabetic nephropathy. LncRNAs can induce SNP events, such as those in mRNA isoforms with extended 3′‐UTRs in the CEP162 transcript, through APA splice site selection.

Functional studies reveal conserved and species‐specific roles: (1) Human: CEP162 mutations (e.g., truncations in CC3) mislocalize TZ proteins (CEP290, RPGRIP1L), causing retinal degeneration and sperm flagellar defects. SNP rs9362054 links CEP162 to diabetic retinopathy.^27^, ^39^ (2) Mouse: Loss of CEP162 disrupts renal cilia, mimicking PKD. Truncated mutants retain microtubule binding but fail to recruit TZ components, highlighting the necessity of CC3 for basal body localization.^26^ (3) Zebrafish: CEP162 depletion phenocopies human LCA and infertility, with swollen ciliary tips and mislocalized IFT88/ARL13b, validating its role in TZ integrity.^20^ (4) Cross‐species insight: CC1‐CC2 conservation enables CEP162 to stabilize microtubule‐TZ interactions, making it a critical node in ciliopathy pathogenesis.

## 5 CONCLUSION AND PERSPECTIVES

**FIGURE 4:**
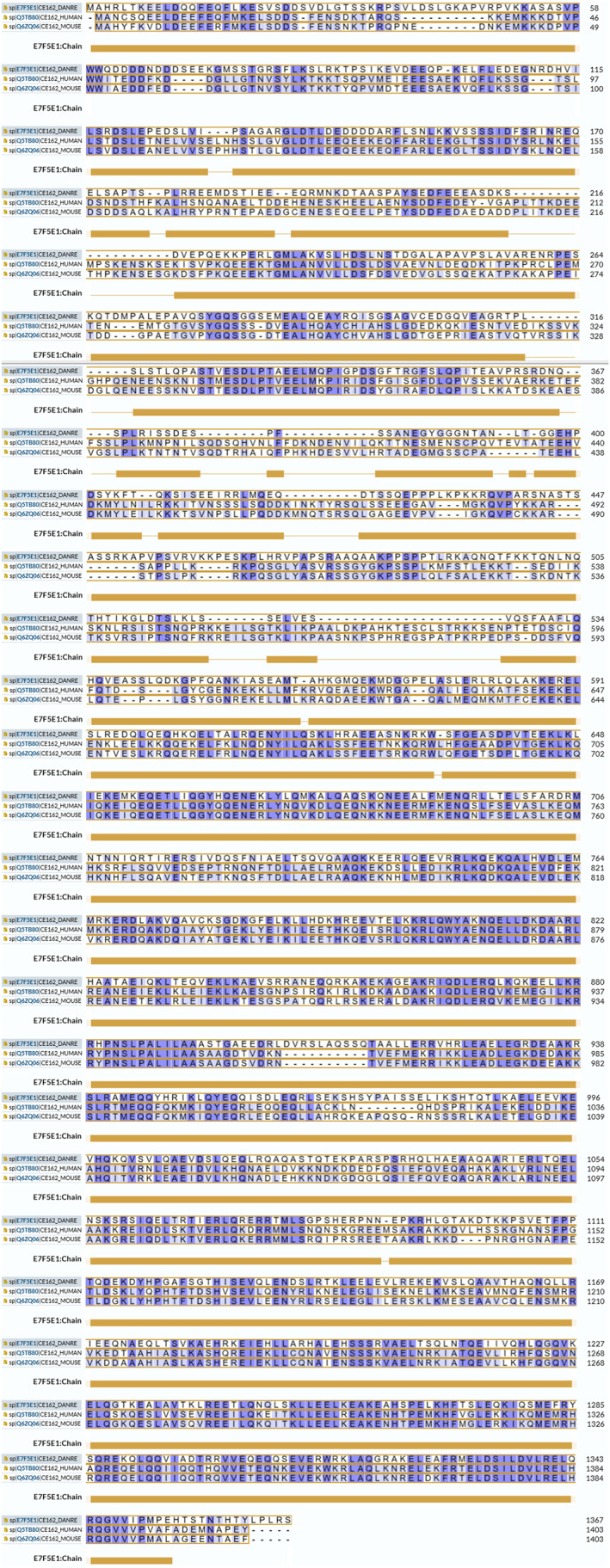
Comparative analysis of CEP162 function across species. This figure integrates sequence conservation and functional studies of CEP162 across human, mouse, and zebrafish models. Sequence alignment diagram (generated using tools such as Clustal Omega) comparing full‐length CEP162 sequences from human (Q5TB80/UniProt), mouse (Q6ZQ06/UniProt), and zebrafish (E7F5E1/UniProt). The alignment is color‐coded to differentiate domains and highlight conservation levels, where dots indicate partial conservation and asterisks denote complete conservation. Gaps in the alignment are represented by dashes (−), optimizing the visualization of similar regions. The yellow bars above the sequence signify predicted domain structures, aiding in the identification of functional segments within the protein. The alignment was performed using the UniProt Align tool (https://www.uniprot.org/align/).^54^

CEP162 interacts with other MAPs to promote TZ assembly at the basal body, serving as a critical ciliary gatekeeper that regulates soluble protein trafficking and maintains cytoplasmic‐ciliary equilibrium. Dysregulation of TZ permeability due to CEP162 abnormalities—including truncating mutations and naturally occurring variants—disrupts IFT, leading to ciliogenesis defects manifesting as retinal degeneration, infertility, and syndromic ciliopathies. As shown in Figure 4, comparative analysis of CEP162 function across species highlights its conserved and species‐specific roles in ciliary biology. Notably, as shown in Table 2, natural CEP162 mutants are closely linked to retinal ciliopathy and diabetic retinopathy, positioning CEP162 as a pivotal therapeutic target for these diseases. Emerging evidence from preclinical and clinical studies (summarized in Table 3) highlights three key translational dimensions.

**TABLE 2:** Evolutionary conservation and phenotypic divergence of CEP162 across species.

Species	Conserved domains	Phenotypic outcomes	Mechanistic insights	Ciliary assembly	Disease model	References
Human	CC1‐CC3 (100%), MT binding (100%)	Retinal degeneration, infertility	CC3 loss→basal body mislocalization→TZ leakage	Truncated CEP162 mutants disrupt TZ assembly, leading to retinal degeneration and infertility.	Associated with Joubert syndrome, diabetic retinopathy (SNP rs9362054)	20, 55
Mouse	CC1‐CC3 (95%), MT binding (98%)	Polycystic kidneys, embryonic lethality	CC3 retention→shortened cilia→renal dysfunction	CEP162 knockout causes embryonic lethality, shortened renal cilia, and polycystic kidney disease.	Models PKD and retinal ciliopathies; rescues neuronal death via truncated CEP162	26
Zebrafish	CC1‐CC2 (82%), MT binding (90%)	Photoreceptor tip swelling, sperm immotility	N‐terminal truncation→TZ mispositioning	CEP162 mutants exhibit retinal degeneration, defective photoreceptor cilia, and impaired sperm motility.	Recapitulates Leber congenital amaurosis (LCA) and infertility phenotypes	20, 32, 41

*Note*: Adapted from references.^20^, ^26^, ^32^, ^41^, ^54^.

**TABLE 3:** Potential therapeutic strategies targeting CEP162.

Strategy	Mechanism of action	Indication	Development stage	Challenges
AAV‐mediated gene therapy	Restoration of wild‐type CEP162 expression	Retinal degeneration, infertility	Preclinical	Germline‐targeting risks
Microtubule stabilizers	Enhanced CEP162‐microtubule binding affinity	Polycystic kidney disease	Phase II clinical trials	Systemic toxicity
PROTAC (proteolysis‐targeting chimera) degraders	Selective degradation of mutant CEP162 (CC3 domain)	CC3 missense mutations	Proof of concept	Tissue‐specific delivery limitations
Nanobody‐based inhibitors	Blockage of pathogenic CC1‐CC2 domain interactions	Ciliopathy‐related complications	Lead optimization	Poor blood–retina barrier penetration

### 5.1 Current therapeutic approaches

As summarized in Table 3, current therapeutic approaches targeting CEP162 include gene replacement (AAV9) and CRISPR/Cas9 exon skipping, which have demonstrated partial functional rescue in retinal and renal models,^56^, ^57^, ^58^ yet domain‐specific precision remains a barrier. PROTAC degraders targeting CC3 mutants (Table 3) exemplify strategies to circumvent global microtubule destabilization.^59^

### 5.2 Diagnostic and prognostic potential

As highlighted in Table 3, urinary exosomal CEP162/CEP290 ratios and AOSLO imaging enable early detection of subclinical ciliary dysfunction, facilitating presymptomatic interventions.^60^, ^61^ Genotype–phenotype correlations (e.g., CC1‐CC2 vs. CC3 mutations) inform personalized therapeutic selection (Table 3).

### 5.3 Research gaps

As outlined in Table 3, nanobody‐based inhibitors (VHH‐162A) and tissue‐targeted LNPs (CD44 binding) may overcome current delivery challenges, as outlined in Table 3.^62^ International registries (e.g., CEP162‐CORE) are critical to harmonize clinical trial endpoints and validate biomarkers across populations.

Critical perspective: Although CEP162‐targeted therapies hold promise, their success hinges on resolving two paradoxes: (1) balancing microtubule stabilization efficacy with off‐tissue toxicity,^15^ and (2) optimizing tissue‐specific delivery to avoid germline or systemic exposure (Table 3). Prioritizing mechanistic studies on CEP162 dosage sensitivity—particularly in retinal and renal tissues—will accelerate the translation of these strategies into clinically viable interventions.

### 5.4 Future directions and clinical applications

Our current insights into CEP162 suggest multiple opportunities for advancing research and clinical interventions: (1) Research priorities: Investigations into the molecular underpinnings of CEP162‐related ciliary dysfunction are paramount. Future study should harness advanced imaging and biochemical techniques to dissect CEP162's interactions within the centrosome and cilium, with a focus on its role in IFT. (2) Therapeutic exploration: The development of targeted therapies for CEP162‐associated ciliopathies is imperative. This includes gene therapy to ameliorate or correct CEP162 mutations, pharmacological chaperones to enhance protein functionality, and the identification of small molecules that can modulate CEP162's activity. (3) Emerging technologies: Innovative technologies such as CRISPR/Cas9 gene editing present promising avenues for both investigating CEP162's role and amending pathogenic mutations. Bioinformatics and computational biology will also be pivotal in uncovering novel interactors and predicting disease mutations, whereas high‐throughput screening will aid in discovering therapeutic agents.

In summary, despite progress, there is a substantial agenda for future research on CEP162. By focusing on these strategic directions, we can expedite the transition from basic research to tangible clinical benefits for ciliopathies associated with CEP162 dysregulation.

## AUTHOR CONTRIBUTIONS

Jun Yin, Jialian Bai, Xiaochong He, Wenjuan He, Hongming Miao, Mengjie Zhang, Zhongying Yu, Bing Ni. Jun Yin, Jialian Bai, Xiaochong He, and Wenjuan He proposed the conceptualization. Jun Yin, Hongming Miao, and Mengjie Zhang wrote and edited the review. Bing Ni, and Zhongying Yu wrote the original draft.

## CONFLICT OF INTEREST STATEMENT

The authors declare no conflict of interest.

## ETHICS STATEMENT

This study was conducted in strict accordance with the guidelines for the care and use of laboratory animals as outlined by the National Institutes of Health (NIH) Guide for the Care and Use of Laboratory Animals. The study protocol was approved by the institutional animal care and use committee at Army Medical University (Approval Number: AMUWEC20230266).

## Data Availability

Data sharing is not applicable to this article as no new data were created or analyzed in this study.

## References

[1] Vasquez‐Limeta, A. , and J. Loncarek . 2021. “Human Centrosome Organization and Function in Interphase and Mitosis.” Seminars in Cell & Developmental Biology 117: 30–41. 10.1016/j.semcdb.2021.03.020. 33836946 PMC8465925

[2] Bettencourt‐Dias, M. , F. Hildebrandt , D. Pellman , G. Woods , and S. A. Godinho . 2011. “Centrosomes and Cilia in Human Disease.” Trends in Genetics 27(8): 307–315. 10.1016/j.tig.2011.05.004. 21680046 PMC3144269

[3] Lloyd, C. , J. Chan , and P. J. Hussey . 2018. “Microtubules and Microtubule‐Associated Proteins.” Cold Spring Harbor Perspectives in Biology 10(6): 3–31. 10.1002/9781119312994.apr0091. PMC5983186 29858272

[4] Bodakuntla, S. , A. S. Jijumon , C. Villablanca , C. Gonzalez‐Billault , and C. Janke . 2019. “Microtubule‐Associated Proteins: Structuring the Cytoskeleton.” Trends in Cell Biology 29(10): 804–819. 10.1016/j.tcb.2019.07.004. 31416684

[5] Camargo Ortega, G. , S. Falk , P. A. Johansson , E. Peyre , L. Broix , S. K. Sahu , W. Hirst , et al. 2019. “The Centrosome Protein AKNA Regulates Neurogenesis via Microtubule Organization.” Nature 567(7746): 113–117. 10.1038/s41586-019-0962-4. 30787442

[6] Coquand, L. , G. S. Victoria , A. Tata , J. A. Carpentieri , J.‐B. Brault , F. Guimiot , V. Fraisier , and A. D. Baffet . 2021. “CAMSAPs Organize an Acentrosomal Microtubule Network from Basal Varicosities in Radial Glial Cells.” The Journal of Cell Biology 220(8). 10.1083/jcb.202003151. PMC8144914 34019079

[7] Batman, U. , J. Deretic , and E. N. Firat‐Karalar . 2022. “The Ciliopathy Protein CCDC66 Controls Mitotic Progression and Cytokinesis by Promoting Microtubule Nucleation and Organization.” PLoS Biology 20(7): e3001708. 10.1371/journal.pbio.3001708. 35849559 PMC9333452

[8] Liu, P. , M. Würtz , E. Zupa , S. Pfeffer , and E. Schiebel . 2021. “Microtubule Nucleation: The Waltz between Gamma‐Tubulin Ring Complex and Associated Proteins.” Current Opinion in Cell Biology 68: 124–131. 10.1016/j.ceb.2020.10.004. 33190097

[9] Monteiro, P. , B. Yeon , S. S. Wallis , and S. A. Godinho . 2023. “Centrosome Amplification Fine Tunes Tubulin Acetylation to Differentially Control Intracellular Organization.” EMBO Journal 42(16): e112812. 10.15252/embj.2022112812. 37403793 PMC10425843

[10] Jiang, L. , W. Lin , C. Zhang , P. E. A. Ash , M. Verma , J. Kwan , E. van Vliet , et al. 2021. “Interaction of Tau with HNRNPA2B1 and N(6)‐methyladenosine RNA Mediates the Progression of Tauopathy.” Molecular Cell 81(20): 4209–4227. 10.1016/j.molcel.2021.07.038. 34453888 PMC8541906

[11] Genova, M. , L. Grycova , V. Puttrich , M. M. Magiera , Z. Lansky , C. Janke , and M. Braun . 2023. “Tubulin Polyglutamylation Differentially Regulates Microtubule‐Interacting Proteins.” EMBO Journal 42(5): e112101. 10.15252/embj.2022112101. 36636822 PMC9975938

[12] Mori, Y. , Y. Inoue , S. Tanaka , S. Doda , S. Yamanaka , H. Fukuchi , and Y. Terada . 2015. “Cep169, a Novel Microtubule Plus‐End‐Tracking Centrosomal Protein, Binds to CDK5RAP2 and Regulates Microtubule Stability.” PLoS One 10(10): e0140968. 10.1371/journal.pone.0140968. 26485573 PMC4613824

[13] Nithianantham, S. , S. Le , E. Seto , W. Jia , J. Leary , K. D. Corbett , J. K. Moore , and J. Al‐Bassam . 2015. “Tubulin Cofactors and Arl2 Are Cage‐like Chaperones that Regulate the Soluble Alphabeta‐Tubulin Pool for Microtubule Dynamics.” Elife 4. 10.7554/elife.08811. PMC4574351 26208336

[14] Srivastava, S. , and D. Panda . 2018. “A Centrosomal Protein STARD9 Promotes Microtubule Stability and Regulates Spindle Microtubule Dynamics.” Cell Cycle 17(16): 2052–2068. 10.1080/15384101.2018.1513764. 30160609 PMC6260213

[15] Mahserejian, S. M. , J. P. Scripture , A. J. Mauro , E. J. Lawrence , E. M. Jonasson , K. S. Murray , J. Li , et al. 2022. “Quantification of Microtubule Stutters: Dynamic Instability Behaviors that Are Strongly Associated with Catastrophe.” Molecular Biology of the Cell 33(3): ar22. 10.1091/mbc.e20-06-0348. 35108073 PMC9250389

[16] Feng, S. , S. Yuan , B. Hou , Z. Liu , Y. Xu , S. Hao , and Y. Lu . 2023. “CEP20 Promotes Invasion and Metastasis of Non‐small Cell Lung Cancer Cells by Depolymerizing Microtubules.” Scientific Reports 13(1): 17484. 10.1038/s41598-023-44754-8. 37838783 PMC10576744

[17] Wu, Z. , H. Chen , Y. Zhang , Y. Wang , Q. Wang , C. Augière , Y. Hou , et al. 2024. “Cep131‐Cep162 and Cby‐Fam92 Complexes Cooperatively Maintain Cep290 at the Basal Body and Contribute to Ciliogenesis Initiation.” PLoS Biology 22(3): e3002330. 10.1371/journal.pbio.3002330. 38442096 PMC10914257

[18] Lapart, J.‐A. , M. Gottardo , E. Cortier , J.‐L. Duteyrat , C. Augière , A. Mangé , J. Jerber , et al. 2019. “Dzip1 and Fam92 Form a Ciliary Transition Zone Complex with Cell Type Specific Roles in Drosophila.” Elife 8. 10.7554/elife.49307. PMC6904220 31821146

[19] Szebenyi, G. , B. Hall , R. Yu , A. I. Hashim , and H. Krämer . 2007. “Hook2 Localizes to the Centrosome, Binds Directly to centriolin/CEP110 and Contributes to Centrosomal Function.” Traffic 8(1): 32–46. 10.1111/j.1600-0854.2006.00511.x. 17140400

[20] Wang, W.‐J. , H. G. Tay , R. Soni , G. S. Perumal , M. G. Goll , F. P. Macaluso , J. M. Asara , J. D. Amack , and M.‐F. Bryan Tsou . 2013. “CEP162 Is an Axoneme‐Recognition Protein Promoting Ciliary Transition Zone Assembly at the Cilia Base.” Nature Cell Biology 15(6): 591–601. 10.1038/ncb2739. 23644468 PMC3815462

[21] Rai, D. , Y. Song , S. Hua , K. Stecker , J. L. Monster , V. Yin , R. Stucchi , et al. 2024. “CAMSAPs and Nucleation‐Promoting Factors Control Microtubule Release from Gamma‐TuRC.” Nature Cell Biology 26(3): 404–420. 10.1038/s41556-024-01366-2. 38424271 PMC10940162

[22] Ping, Y. , K. Ohata , K. Kikushima , T. Sakamoto , A. Islam , L. Xu , H. Zhang , et al. 2023. “Tubulin Polyglutamylation by TTLL1 and TTLL7 Regulate Glutamate Concentration in the Mice Brain.” Biomolecules 13(5): 784. 10.3390/biom13050784. 37238654 PMC10216738

[23] Kumari, A. , and D. Panda . 2018. “Regulation of Microtubule Stability by Centrosomal Proteins.” IUBMB Life 70(7): 602–611. 10.1002/iub.1865. 29734495

[24] Kuo, Y.‐W. , and J. Howard . 2021. “Cutting, Amplifying, and Aligning Microtubules with Severing Enzymes.” Trends in Cell Biology 31(1): 50–61. 10.1016/j.tcb.2020.10.004. 33183955 PMC7749064

[25] Chen, Y. , and W. O. Hancock . 2015. “Kinesin‐5 Is a Microtubule Polymerase.” Nature Communications 6(1): 8160. 10.1038/ncomms9160. PMC4600729 26437877

[26] Nuzhat, N. , K. Van Schil , S. Liakopoulos , M. Bauwens , A. D. Rey , Stephan Käseberg , M. Jäger , et al. 2023. “CEP162 Deficiency Causes Human Retinal Degeneration and Reveals a Dual Role in Ciliogenesis and Neurogenesis.” Journal of Clinical Investigation 133(8). 10.1172/jci161156. PMC10104899 36862503

[27] Li, H. , M. Ning , Q. Li , T. Wang , W. Li , J. Xiao , L. Wang , et al. 2023. “The Association of Five Polymorphisms with Diabetic Retinopathy in a Chinese Population.” Ophthalmic Genetics 44(4): 346–351. 10.1080/13816810.2023.2194494. 37066695

[28] Grey, C. , and B. de Massy . 2021. “Chromosome Organization in Early Meiotic Prophase.” Frontiers in Cell and Developmental Biology 9: 688878. 10.3389/fcell.2021.688878. 34150782 PMC8209517

[29] Leon, A. , B. Omri , A. Gely , C. Klein , and P. Crisanti . 2006. “QN1/KIAA1009: a New Essential Protein for Chromosome Segregation and Mitotic Spindle Assembly.” Oncogene 25(13): 1887–1895. 10.1038/sj.onc.1209215. 16302001

[30] Basiri, M. L. , A. Ha , A. Chadha , N. M. Clark , A. Polyanovsky , B. Cook , and T. Avidor‐Reiss . 2014. “A Migrating Ciliary Gate Compartmentalizes the Site of Axoneme Assembly in Drosophila Spermatids.” Current Biology 24(22): 2622–2631. 10.1016/j.cub.2014.09.047. 25447994 PMC4254545

[31] Fagerberg, L. , B. M. Hallström , P. Oksvold , C. Kampf , D. Djureinovic , J. Odeberg , M. Habuka , et al. 2014. “Analysis of the Human Tissue‐specific Expression by Genome‐wide Integration of Transcriptomics and Antibody‐Based Proteomics.” Molecular & Cellular Proteomics 13(2): 397–406. 10.1074/mcp.m113.035600. 24309898 PMC3916642

[32] Won, J. , C. M. de Evsikova , R. S. Smith , W. L. Hicks , M. M. Edwards , C. Longo‐Guess , T. Li , J. K. Naggert , and P. M. Nishina . 2011. “NPHP4 Is Necessary for Normal Photoreceptor Ribbon Synapse Maintenance and Outer Segment Formation, and for Sperm Development.” Human Molecular Genetics 20(3): 482–496. 10.1093/hmg/ddq494. 21078623 PMC3016909

[33] Avidor‐Reiss, T. , A. Ha , and M. L. Basiri . 2017. “Transition Zone Migration: A Mechanism for Cytoplasmic Ciliogenesis and Postaxonemal Centriole Elongation.” Cold Spring Harbor Perspectives in Biology 9(8): a028142. 10.1101/cshperspect.a028142. 28108487 PMC5538411

[34] Jana, S. C. , S. Mendonça , P. Machado , S. Werner , J. Rocha , A. Pereira , H. Maiato , and M. Bettencourt‐Dias . 2018. “Differential Regulation of Transition Zone and Centriole Proteins Contributes to Ciliary Base Diversity.” Nature Cell Biology 20(8): 928–941. 10.1038/s41556-018-0132-1. 30013109

[35] Wiegering, A. , R. Dildrop , L. Kalfhues , A. Spychala , S. Kuschel , J. M. Lier , T. Zobel , et al. 2018. “Cell Type‐specific Regulation of Ciliary Transition Zone Assembly in Vertebrates.” EMBO Journal 37(10). 10.15252/embj.201797791. PMC5978567 29650680

[36] Persico, V. , G. Callaini , and M. G. Riparbelli . 2019. “The Microtubule‐Depolymerizing Kinesin‐13 Klp10A Is Enriched in the Transition Zone of the Ciliary Structures of Drosophila melanogaster.” Frontiers in Cell and Developmental Biology 7: 173. 10.3389/fcell.2019.00173. 31497602 PMC6713071

[37] Udupa, P. , and D. K. Ghosh . 2024. “The Emerging Functions of Intraflagellar Transport 52 in Ciliary Transport and Ciliopathies.” Traffic 25(1): e12929. 10.1111/tra.12929. 38272449

[38] Vitre, B. , A. Guesdon , and B. Delaval . 2020. “Non‐ciliary Roles of IFT Proteins in Cell Division and Polycystic Kidney Diseases.” Frontiers in Cell and Developmental Biology 8: 578239. 10.3389/fcell.2020.578239. 33072760 PMC7536321

[39] Park, K. , and M. R. Leroux . 2022. “Composition, Organization and Mechanisms of the Transition Zone, a Gate for the Cilium.” EMBO Reports 23(12): e55420. 10.15252/embr.202255420. 36408840 PMC9724682

[40] Dean, S. , F. Moreira‐Leite , V. Varga , and K. Gull . 2016. “Cilium Transition Zone Proteome Reveals Compartmentalization and Differential Dynamics of Ciliopathy Complexes.” Proceedings of the National Academy of Sciences of the U S A 113(35): E5135–E5143. 10.1073/pnas.1604258113. PMC5024643 27519801

[41] Sang, L. , J. J. Miller , K. C. Corbit , R. H. Giles , M. J. Brauer , E. A. Otto , L. M. Baye , et al. 2011. “Mapping the NPHP‐JBTS‐MKS Protein Network Reveals Ciliopathy Disease Genes and Pathways.” Cell 145(4): 513–528. 10.1016/j.cell.2011.04.019. 21565611 PMC3383065

[42] Awata, T. , H. Yamashita , S. Kurihara , T. Morita‐Ohkubo , Y. Miyashita , S. Katayama , K. Mori , et al. 2014. “A Genome‐wide Association Study for Diabetic Retinopathy in a Japanese Population: Potential Association with a Long Intergenic Non‐coding RNA.” PLoS One 9(11): e111715. 10.1371/journal.pone.0111715. 25364816 PMC4218806

[43] Wang, J. , H. R. Thomas , R. G. Thompson , S. C. Waldrep , J. Fogerty , P. Song , Z. Li , et al. 2022. “Variable Phenotypes and Penetrance between and within Different Zebrafish Ciliary Transition Zone Mutants.” Dis Model Mech 15(12). 10.1242/dmm.049568. PMC9844136 36533556

[44] Zhou, L. , H. Liu , S. Liu , X. Yang , Y. Dong , Y. Pan , Z. Xiao , et al. 2023. “Structures of Sperm Flagellar Doublet Microtubules Expand the Genetic Spectrum of Male Infertility.” Cell 186(13): 2897–2910. 10.1016/j.cell.2023.05.009. 37295417

[45] Leung, M. R. , J. Zeng , X. Wang , M. C. Roelofs , W. Huang , R. Zenezini Chiozzi , J. F. Hevler , et al. 2023. “Structural Specializations of the Sperm Tail.” Cell 186(13): 2880–2896. 10.1016/j.cell.2023.05.026. 37327785 PMC10948200

[46] Hall, E. A. , M. Keighren , M. J. Ford , T. Davey , A. P. Jarman , L. B. Smith , I. J. Jackson , and P. Mill . 2013. “Acute versus Chronic Loss of Mammalian Azi1/Cep131 Results in Distinct Ciliary Phenotypes.” PLoS Genetics 9(12): e1003928. 10.1371/journal.pgen.1003928. 24415959 PMC3887133

[47] Lv, M. , W. Liu , W. Chi , X. Ni , J. Wang , H. Cheng , W.‐Y. Li , et al. 2020. “Homozygous Mutations in DZIP1 Can Induce Asthenoteratospermia with Severe MMAF.” Journal of Medical Genetics 57(7): 445–453. 10.1136/jmedgenet-2019-106479. 32051257 PMC7361034

[48] Huang, N. , D. Zhang , F. Li , P. Chai , S. Wang , J. Teng , and J. Chen . 2018. “M‐phase Phosphoprotein 9 Regulates Ciliogenesis by Modulating CP110‐CEP97 Complex Localization at the Mother Centriole.” Nature Communications 9(1): 4511. 10.1038/s41467-018-06990-9. PMC6207757 30375385

[49] Szymanska, K. , I. Berry , C. V. Logan , S. R. R. Cousins , H. Lindsay , H. Jafri , Y. Raashid , et al. 2012. “Founder Mutations and Genotype‐Phenotype Correlations in Meckel‐Gruber Syndrome and Associated Ciliopathies.” Cilia 1(1): 18. 10.1186/2046-2530-1-18. 23351400 PMC3579735

[50] Zhang, Y. , S. Seo , S. Bhattarai , K. Bugge , C. C. Searby , Q. Zhang , A.V. Drack , E. M. Stone , and V. C. Sheffield . 2014. “BBS Mutations Modify Phenotypic Expression of CEP290‐Related Ciliopathies.” Human Molecular Genetics 23(1): 40–51. 10.1093/hmg/ddt394. 23943788 PMC3857943

[51] Liu, Y. , X. Liu , C. Lin , X. Jia , H. Zhu , J. Song , and Y. Zhang . 2021. “Noncoding RNAs Regulate Alternative Splicing in Cancer.” Journal of Experimental & Clinical Cancer Research 40(1): 11. 10.1186/s13046-020-01798-2. 33407694 PMC7789004

[52] Azzam, S. K. , W. M. Osman , S. Lee , K. Khalaf , A. H. Khandoker , W. Almahmeed , H. F. Jelinek , and H. S. Al Safar . 2019. “Genetic Associations with Diabetic Retinopathy and Coronary Artery Disease in Emirati Patients with Type‐2 Diabetes Mellitus.” Frontiers in Endocrinology 10: 283. 10.3389/fendo.2019.00283. 31130920 PMC6509200

[53] Liu, M. , J. Chen , C. Zhang , S. Liu , X. Chao , H. Yang , A. Muhammad , B. Zhou , W. Ao , and A. P. Schinckel . 2024. “Deciphering Estrus Expression in Gilts: The Role of Alternative Polyadenylation and LincRNAs in Reproductive Transcriptomics.” Animals 14(5): 791. 10.3390/ani14050791. 38473176 PMC10931002

[54] Zaru, R. , S. Orchard , and C. UniProt . 2023. “UniProt Tools: BLAST, Align, Peptide Search, and ID Mapping.” Curr Protoc 3(3): e697. 10.1002/cpz1.697. 36943033 PMC10034637

[55] Li, H. , M. Ning , Q. Li , T. Wang , W. Li , J. Xiao , L. Wang , et al. 2023. “The Association of Five Polymorphisms with Diabetic Retinopathy in a Chinese Population.” Ophthalmic Genetics 44(4): 346–351. 10.1080/13816810.2023.2194494. 37066695

[56] Jacobson, S. G. , A. V. Cideciyan , R. Ratnakaram , E. Heon , S. B. Schwartz , A. J. Roman , M. C. Peden , et al. 2012. “Gene Therapy for Leber Congenital Amaurosis Caused by RPE65 Mutations: Safety and Efficacy in 15 Children and Adults Followed up to 3 Years.” Archives of Ophthalmology 130(1): 9–24. 10.1001/archophthalmol.2011.298. 21911650 PMC3600816

[57] Dalkara, D. , L. C. Byrne , R. R. Klimczak , M. Visel , L. Yin , W. H. Merigan , J. G. Flannery , and D. V. Schaffer . 2013. “In Vivo‐directed Evolution of a New Adeno‐Associated Virus for Therapeutic Outer Retinal Gene Delivery from the Vitreous.” Science Translational Medicine 5(189): 189ra76. 10.1126/scitranslmed.3005708. 23761039

[58] Long, C. , L. Amoasii , A. A. Mireault , J. R. McAnally , H. Li , E. Sanchez‐Ortiz , S. Bhattacharyya , J. M. Shelton , R. Bassel‐Duby , and E. N. Olson . 2016. “Postnatal Genome Editing Partially Restores Dystrophin Expression in a Mouse Model of Muscular Dystrophy.” Science 351(6271): 400–403. 10.1126/science.aad5725. 26721683 PMC4760628

[59] Bondeson, D. P. , A. Mares , I. E. D. Smith , E. Ko , S. Campos , A. H. Miah , K. E. Mulholland , et al. 2015. “Catalytic In Vivo Protein Knockdown by Small‐Molecule PROTACs.” Nature Chemical Biology 11(8): 611–617. 10.1038/nchembio.1858. 26075522 PMC4629852

[60] Cao, J.‐Y. , B. Wang , T.‐T. Tang , Y. Wen , Z.‐L. Li , S.‐T. Feng , M. Wu , et al. 2021. “Exosomal miR‐125b‐5p Deriving from Mesenchymal Stem Cells Promotes Tubular Repair by Suppression of P53 in Ischemic Acute Kidney Injury.” Theranostics 11(11): 5248–5266. 10.7150/thno.54550. 33859745 PMC8039965

[61] Roosing, S. , M. Romani , M. Isrie , R. O. Rosti , A. Micalizzi , D. Musaev , T. Mazza , et al. 2016. “Mutations in CEP120 Cause Joubert Syndrome as Well as Complex Ciliopathy Phenotypes.” Journal of Medical Genetics 53(9): 608–615. 10.1136/jmedgenet-2016-103832. 27208211 PMC5013089

[62] Cheng, Q. , T. Wei , L. Farbiak , L. T. Johnson , S. A. Dilliard , and D. J. Siegwart . 2020. “Selective Organ Targeting (SORT) Nanoparticles for Tissue‐specific mRNA Delivery and CRISPR‐Cas Gene Editing.” Nature Nanotechnology 15(4): 313–320. 10.1038/s41565-020-0669-6. PMC7735425 32251383

